# Z-FL-COCHO, a cathepsin S inhibitor, enhances oxaliplatin-mediated apoptosis through the induction of endoplasmic reticulum stress

**DOI:** 10.1038/s12276-018-0138-6

**Published:** 2018-08-17

**Authors:** Seung Un Seo, Kyoung-jin Min, Seon Min Woo, Taeg Kyu Kwon

**Affiliations:** Department of Immunology, School of Medicine, Keimyung University, Daegu, Korea

## Abstract

Multiple cancer cells highly express cathepsin S, which has pro-tumoral effects. However, it was previously unknown whether knockdown or a pharmacological inhibitor (ZFL) of cathepsin S acts as an inducer of ER stress. Here, ZFL and knockdown of cathepsin S markedly induced ER stress through the up-regulation of calcium levels in the cytosol. Induction of calcium levels by inhibition of cathepsin S is markedly blocked by an inhibitor of the IP3 receptor and the ryanodine receptor Ca^2+^ channel in the ER, but an inhibitor of a mitochondrial Ca^2+^ uniporter had no effect on ZFL-induced calcium levels. Furthermore, production of mitochondrial ROS by ZFL was associated with an increase in cytosolic calcium levels. ZFL-mediated ER stress enhanced anti-cancer drug-induced apoptotic cell death, and pretreatment with chemical chaperones or down-regulation of ATF4 and CHOP by small interfering RNA markedly reduced ZFL plus oxaliplatin-induced apoptosis. Taken together, our findings reveal that inhibition of cathepsin S is an inducer of ER stress; these findings may contribute to the enhancement of therapeutic efficiency in cancer cells.

## Cancer: Enhancing sensitivity to anti-cancer drugs

A drug that inhibits a key cancer enzyme could be used in combination with anti-cancer drugs to improve sensitivity to treatment. The intracellular endoplasmic reticulum (ER) is involved in several vital processes in cells, including folding and processing proteins. Taeg Kyu Kwon at Keimyung University, Daegu, South Korea, and co-workers have demonstrated how inhibition of cathepsin S, which is expressed in many cancer cells, induces ER stress. In trials on human kidney cancer cells grafted onto mice and in vitro, the team found that ZFL (cathepsin S inhibitor) triggered transient ER stress by increasing calcium levels inside cells. Subsequent treatment with the anti-cancer drug oxaliplatin resulted in increased cancer cell death.

## Introduction

Cathepsin S is a lysosomal cysteine protease highly expressed in antigen-presenting cells (B cells, macrophages, microglia, and dendritic cells)^1–4^. The main function of this protease is the degradation of the class II major histocompatibility complex-associated invariant chain, which is related to the immune response^4^. However, cathepsin S is also detected in malignant cells^5–7^, and many researchers have suggested the pro-tumoral effects of cathepsin S in cancer cells. For example, inhibition of cathepsin S induces apoptosis in nasopharyngeal carcinoma^8,9^, glioma^10^, and hepatocellular carcinoma^11^ and inhibits invasion and angiogenesis in hepatocellular carcinoma^12^. Furthermore, cathepsin S plays critical roles in tumor development. Cathepsin S-null (cathepsin S^−/−^) mice crossed with the spontaneous pancreatic beta-cell carcinogenesis model (RIP1-Tag2) exhibited impaired tumor growth and angiogenesis^13^. In addition, the expression level of cathepsin S is related to poor outcomes in glioblastoma^14^, lung cancer^15^, and colorectal cancer^16^. Inhibitor of cathepsin S has a synergistic effect with chemotherapeutic drugs. For example, combined treatment with Fsn0503 (a cathepsin S inhibitory antibody) and an anti-vascular endothelial growth factor antibody exhibits a synergistic inhibitory effect of angiogenesis in the tumor microenvironment^17^. Fsn0503 also enhanced the anti-cancer effect of CPT-11 in colorectal cancer^18^, and Z-FL-COCHO (ZFL; a cathepsin S inhibitor) sensitized TRAIL (tumor necrosis factor-related apoptosis-inducing ligand)-mediated apoptosis in renal carcinoma cells^19^. Therefore, cathepsin S is a promising therapeutic target for treating cancer.

The endoplasmic reticulum (ER) is responsible for protein folding, translocation, and post-translational modification in cells. However, disturbance of the ER environment by intra- or extra-cellular stimuli are detected by ER sensor proteins (IRE1α (inositol requiring enzyme/endonuclease 1), ATF6 (activating transcription factor 6), and PERK (double stranded RNA-activated protein kinase (PKR)-like ER kinase)), resulting in the induction of ER stress^20^. To overcome such ER stress, cells turn on the unfolded protein response (UPR) (inhibition of protein translation, degradation of misfolded proteins, and production of molecular chaperones); however, if the UPR is not sufficient to reduce ER stress, cells undergo cell death^21^. Activation of PERK by severe and prolonged ER stress globally inhibits new protein synthesis and increases the translation of selected messenger RNAs (mRNAs), including ATF4 (activating transcription factor 4). Up-regulated ATF4 as a transcription factor increases the expression of CHOP (CCAAT-enhancer-binding protein homologous protein) as well as the expression of multiple proteins to recover the cell status and adapt to ER stress^21^. The up-regulation of CHOP expression has critical roles in ER stress-induced apoptosis. Mouse embryonic fibroblasts derived from Chop^−/−^ animals exhibite less induction of cell death by tunicamycin-induced ER stress, compared with wild type^22^, and multiple drugs induce ER stress-mediated apoptosis through the up-regulation of CHOP expression^23–26^. In addition, up-regulation of CHOP has been shown to enhance the sensitivity of anti-cancer drug-induced cell death^27–29^.

In the current study, we investigated the effect of cathepsin S inhibition on ER stress as well as the molecular mechanisms underlying cathepsin S inhibition-induced ER stress in human renal carcinoma cells.

## Materials and methods

### Cell culture and materials

American Type Culture Collection supplied all human cancer cells (renal carcinoma: Caki, ACHN, and A498, lung carcinoma: A549, breast carcinoma: MDA-MB-231) and mouse kidney cells (TCMK-1) (Manassas, VA, USA). Normal human mesangial cells were purchased from Lonza (CC-2559, Basel, Switzerland). Cells were grown in Dulbecco's modified Eagle's medium or RPMI supplemented with 10% fetal bovine serum and 100 μg/mL gentamycin. All cell lines were tested for mycoplasma contamination. The cell lines were authenticated by standard morphologic examination using microscopy. R&D Systems supplied z-VAD-fmk and tumor necrosis factor-α (TNF-α; Minneapolis, MN, USA), and Calbiochem supplied *N*-acetyl-l-cysteine (NAC), Z-FL-COCHO (ZFL), Trolox, and 2-aminoethoxydiphenyl borate (APB) (San Diego, CA, USA). pEGFP-HSP70 was a gift from Lois Greene (Addgene plasmid # 15215)^30^. Santa Cruz Biotechnology supplied sorafenib, anti-cathepsin S, anti-ATF4, and anti-HSP70 antibodies and small interfering RNA (siRNA; cathepsin S, ATF4, and CHOP), and Cell Signaling Technology supplied anti-PARP, anti-CHOP, anti-REDD1, and anti-cleaved caspase-3 antibodies (Beverly, MA, USA). Enzo Life Science supplied cisplatin, anti-GRP78, and anti-pro-caspase-3 antibodies (Farmington, NY, USA). The doxorubicin was purchased from Tocris Bioscience (Minneapolis, MN, USA). EDM Millipore supplied anti-Fas antibody (human, activating) clone CH11 (05–201) (EMD Millipore, Darmstadt, Germany), and Cayman Chemical supplied gefitinib (Ann Arbor, MI, USA). Bioneer supplied the green fluorescent protein (GFP; control) siRNA (Daejeon, Korea). Sigma Chemical Co. supplied other reagents used in our study (St. Louis, MO, USA).

### Western blot analysis and flow cytometry analysis

Whole-cell lysates were obtained as described previously using modified RIPA buffer^31–33^. We performed the western blotting and flow cytometry analysis as described in our previous study^34^.

### Intracellular Ca^2+^ detection

Cells were harvested and resuspended in phosphate-buffered saline (PBS) containing 2 μM Fluo-4/AM (Molecular Probes, Invitrogen) for 45 min in an incubator with frequent agitation. The cells were then resuspended in PBS for FACS acquisition (BD Biosciences, San Diego, CA, USA).

### DAPI staining and DNA fragmentation assay

For 4′,6′-diamidino-2-phenylindole (DAPI) staining and DNA fragmentation, cells were treated with 25 μM oxaliplatin and/or 2 μM ZFL for 24 h. Caki cells were fixed, washed with PBS, and stained with a 300 nM DAPI solution (Roche, Mannheim, Germany), or DNA fragmentation was detected using a cell death detection ELISA plus kit as described in our study (Boehringer Mannheim, Indianapolis, IN, USA)^31^.

### Asp-Glu-Val-Asp-ase (DEVDase) activity assay

Cell were treated with 25 μM oxaliplatin and/or 2 μM ZFL for 24 h, and then 20 μg of cell lysates was incubated with reaction buffer as described in our previous study^31^. We measured caspase activity at 405 nm absorbance using a spectrophotometer.

### Animal experiments

Central Lab Animal Inc. supplied male BALB/c-nude mice (5 weeks) (Seoul, Korea). The IRB Keimyung University Ethics Committee approved our research protocol, and all mice were maintained for 7 days to acclimatize to the surroundings before our experiments (temperature: 25 ± 2 °C, humidity: 55 ± 5%).

### In vivo xenograft model

Caki cells (2 × 10^6^) were subcutaneously grafted onto male BALB/c-nude mice, and after 2 weeks, 14 mice were randomly divided into the vehicle and ZFL groups. ZFL was dissolved in 20% dimethyl sulfoxide and 80% PBS (pH 7.4), and 5 mg/kg ZFL was injected into mice via intraperitoneal (i.p.) injection. Mice were treated with vehicle or ZFL three times per week for 28 days, and the protein was obtained at the time of killing.

### Statistical analysis

Data in our study were analyzed by one-way analysis of variance and post-hoc comparisons (Student–Newman– Keuls) using Statistical Package for Social Sciences 22.0 software (SPSS Inc.; Chicago, IL, USA).

## Results

### Cathepsin S inhibitor induces endoplasmic reticulum stress but not apoptosis

**Fig. 1:**
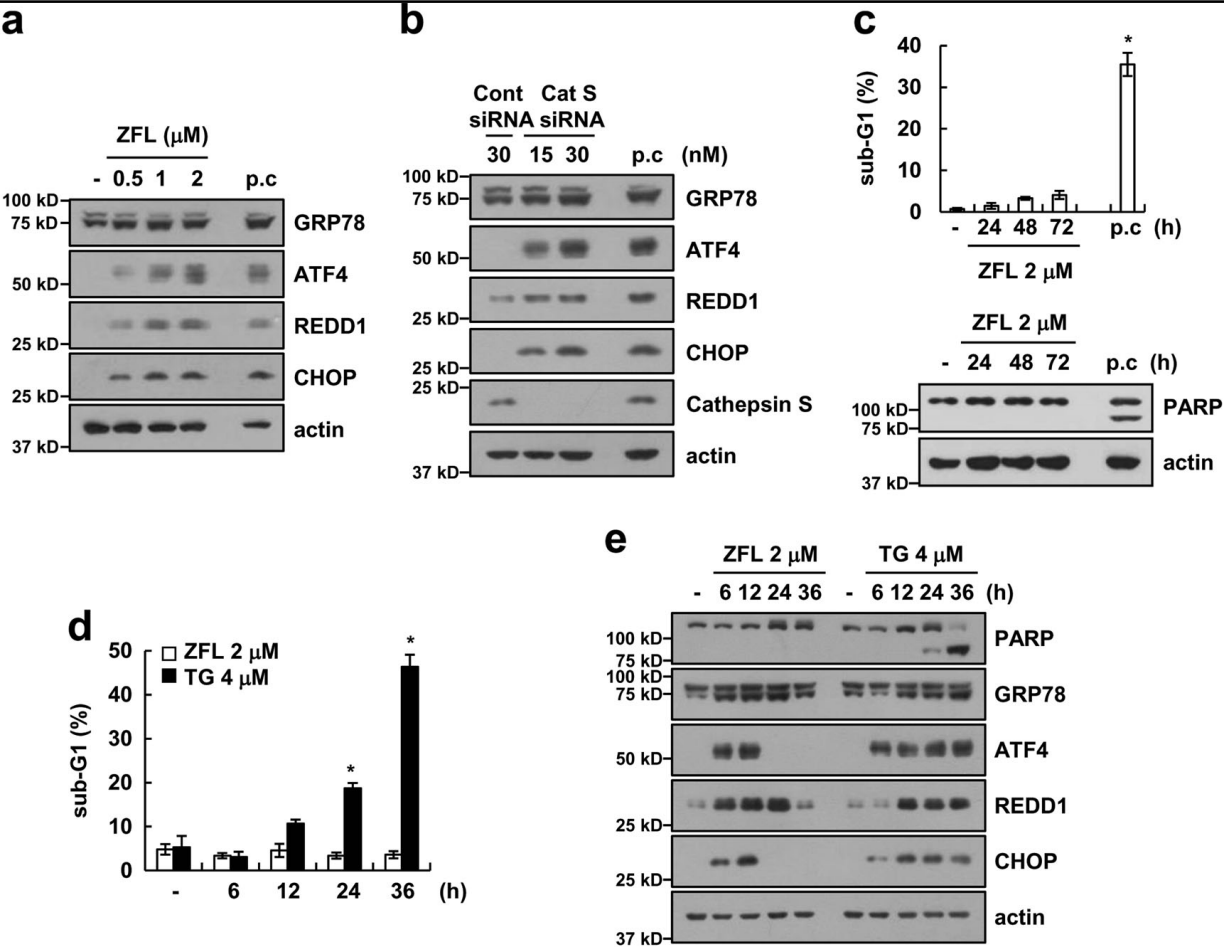
Inhibition of cathepsin S induces ER stress. **a** Human renal carcinoma Caki cells were treated with 0.5, 1, or 2 μM ZFL for 8 h (p.c. positive control; brefeldin A for 8 h). **b** Caki cells were transfected with siRNA against control or cathepsin S for 24 h (p.c.; brefeldin A for 8 h). **c** Caki cells were treated with 2 μM ZFL for 24, 48, or 72 h (p.c.; 20 ng/mL TNF-α plus 5 μg/mL CHX for 24 h). **d**, **e** Caki cells were treated with 2 μM ZFL or 4 μM thapsigargin (TG) for the indicated time periods. Flow cytometry was used to detect the sub-G1 population, and western blotting was used to detect protein levels of PARP, GRP78, ATF4, REDD1, CHOP, and/or actin. The data in (**c**, **d**) are presented as the mean ± SD of three independent samples; *p < 0.01 compared to the control

The molecular mechanism underlying cathepsin S-mediated ER stress induction remains unknown. Therefore, we investigated the effect of a cathepsin S inhibitor (ZFL) on the induction of ER stress. ZFL dose-dependently increased the expression of ER stress marker proteins (GRP78, ATF4, REDD1, and CHOP) (Fig. 1a). We next investigated whether the down-regulation of cathepsin S by siRNA modulates the expression of GRP78, ATF4, REDD1, and CHOP in a manner similar to ZFL treatment. Down-regulation of cathepsin S also induced GRP78, ATF4, REDD1, and CHOP protein expression (Fig. 1b). To determine the effect of ZFL-mediated ER stress on apoptotic cell death, we examined apoptosis in ZFL-treated cells. We found that ZFL induced ER stress responses, but ZFL did not induce poly (ADP-ribose) polymerase (PARP) cleavage or increase the levels of the sub-G1 population, which are markers of apoptotic cell death (Fig. 1c). ER stress is critical for the induction of apoptosis^35^, but ZFL did not induce apoptosis (Fig. 1c). Therefore, we investigated the duration and extent of ER stress in ZFL-treated cells. Thapsigargin, an ER stress inducer, markedly induced apoptosis and maintained the up-regulation of ER stress marker proteins up to 36 h (Fig. 1d, e). In contrast, ZFL transiently induced the expression of ER stress marker proteins, which declined at 24–36 h (Fig. 1d, e).

### Intracellular Ca^2+^ is a key factor in the ZFL-mediated ER stress response

**Fig. 2:**
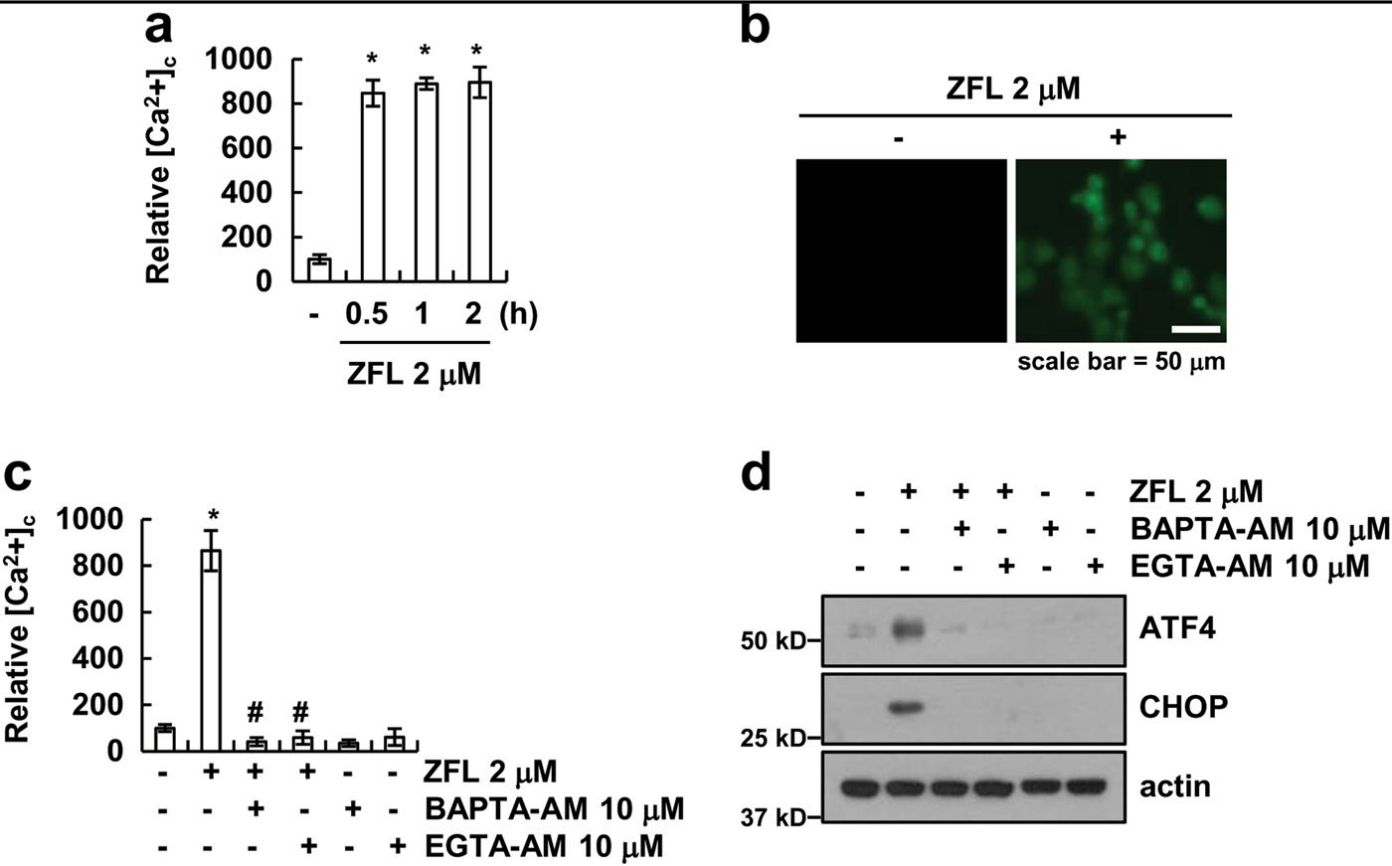
ZFL induces intracellular Ca^2+^ release in Caki cells. **a**, **b** Caki cell were treated with 2 μM ZFL for the indicated time periods (**a**) or 2 h (**b**). After treatment with ZFL, cells were loaded with Flou-4/AM fluorescent dye, and flow cytometry (**a**) or fluorescence microscopy (**b**) was used to detect calcium levels. **c**, **d** Caki cells were pre-treated with 10 μM BAPTA-AM and 10 μM EGTA-AM for 30 min and were then treated with 2 μM ZFL for 2 h (**c**) and 8 h (**d**). Cells were loaded with Fluo-4/AM fluorescent dye, and flow cytometry was used to measurement calcium levels (**c**). Western blotting was used to detect protein levels of ATF4, CHOP, and actin (**d**). The values in (**a**, **c**) represent the mean ± SD of three independent samples; *p < 0.01 compared to the control; ^#^*p* < 0.01 compared to ZFL

Since an imbalance of Ca^2+^ homeostasis is a key factor in ER stress, we examined the possibility that inhibition of cathepsin S modulates intracellular Ca^2+^ levels in Caki cells. We detected intracellular Ca^2+^ levels by flow cytometry and fluorescence microscopy using Fluo-4/AM (a cell-permeable Ca^2+^-indicator dye). As shown in Fig. 2a, Fluo-4/AM fluorescence intensity increased at 30 min of ZFL treatment. We further confirmed the ZFL-induced increase in Fluo-4/AM staining intensity using fluorescence microscopy (Fig. 2b). Next, we investigated the link between ER stress and Ca^2+^ release in ZFL-treated Caki cells. The chelators of Ca^2+^ (EGTA-AM and BAPTA-AM) inhibited not only the up-regulation of intracellular Ca^2+^ levels but also the up-regulation of ATF4 and CHOP protein expression in ZFL-treated Caki cells (Fig. 2c, d). These results reveal that a ZFL-induced increase in intracellular Ca^2+^ levels has a critical role in the induction of ER stress.

### Ca^2+^ release from the ER is critical for the ZFL-mediated induction of the ER stress response

**Fig. 3:**
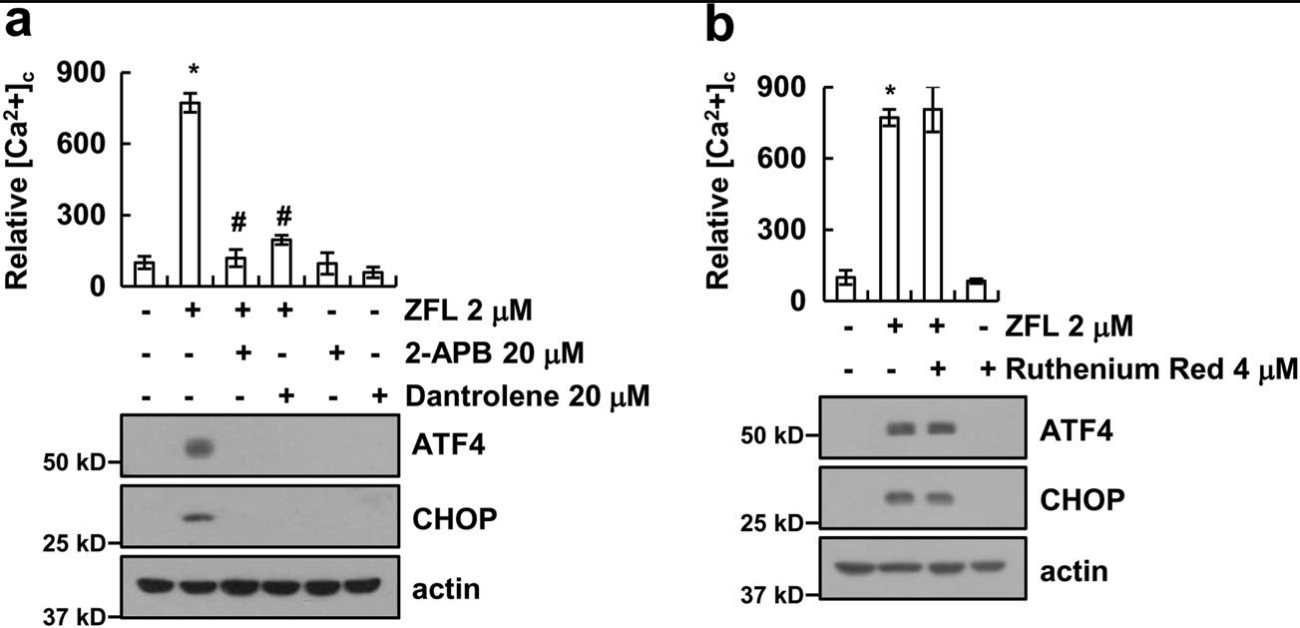
ZFL induces ER stress via calcium release from the ER. **a** Caki cells were pre-treated with 20 μM 2-aminoethoxydiphenyl borate (2-APB) and 20 μM dantrolene for 30 min and then treated with 2 μM ZFL for 2 h (upper panel) or 8 h (lower panel). **b** Caki cells were pre-treated with 4 μM ruthenium red for 30 min and then treated with 2 μM ZFL for 2 h (upper panel) or 8 h (lower panel). Cells were loaded with Fluo-4/AM fluorescent dye, and calcium levels were measured using flow cytometry. Western blotting was used to detect the protein levels of ATF4, CHOP, and actin. The values in (**a**, **b**) represent the mean ± SD of three independent samples; *p < 0.01 compared to control; ^#^*p* < 0.01 compared to ZFL

Since the ER is a primary organelle for calcium storage^36^, we employed specific inhibitors of the inositol 1,4,5-trisphosphate (IP3) receptor (IP3R) and the ryanodine receptor (RyR)^37^, which regulate major Ca^2+^ release channels in the ER. We found that 2-APB (an inhibitor of IP3R)^38^ and dantrolene (an inhibitor of the RyR)^39^ very effectively inhibited the ZFL-induced Ca^2+^ release and the protein expression of ATF4 and CHOP (Fig. 3a). In contrast, ruthenium red (an inhibitor of mitochondrial Ca^2+^ uptake and release)^40,41^ had no effect on Ca^2+^ levels or the expression of ATF4 and CHOP in ZFL-treated cells (Fig. 3b). Collectively, our data reveal that Ca^2+^ release from the ER may play a critical role in the ZFL-mediated induction of ER stress.

### Mitochondrial ROS production is critical for ZFL-induced Ca^2+^ release

**Fig. 4:**
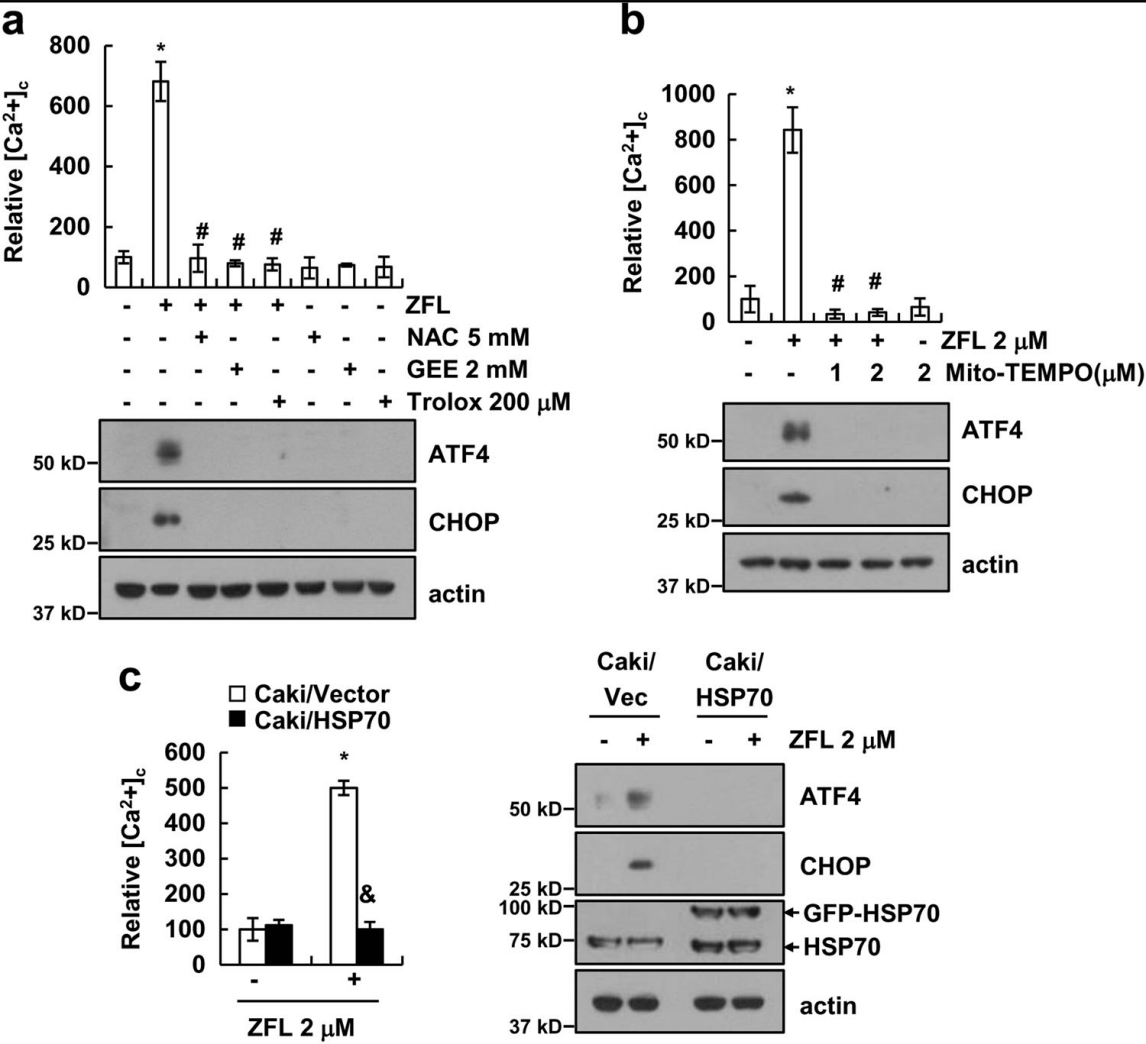
Mitochondrial ROS production by ZFL is involved in the induction of ER stress. **a** Caki cells were pre-treated with NAC, GEE, and Trolox for 30 min and then treated with 2 μM ZFL for 2 h (upper panel) or 8 h (lower panel). **b** Caki cells were pre-treated with indicated concentrations of Mito-TEMPO for 30 min, and then 2 μM ZFL was added for 2 h (left panel) or 8 h (right panel). **c** Caki/Vector and Caki/HSP70 were treated with 2 μM ZFL for 2 h or 8 h. Cells were loaded with Fluo-4/AM, and we detected the calcium levels using flow cytometry. Western blotting was used to detect the protein levels of ATF4, CHOP, HSP70, and/or actin. The values in (**a**–**c**) represent the mean ± SD of three independent samples; *p < 0.01 compared to the control; ^#^*p* < 0.01 compared to ZFL; ^&^*p* < 0.01 compared to ZFL-treated Caki/Vec

Recently, we reported that ZFL induces lysosomal membrane permeability (LMP), which is associated with mitochondrial dysfunction and mitochondrial reactive oxygen species (ROS) production^19^. To investigate the role of ROS in the ZFL-induced Ca^2+^ release, we used ROS scavengers [NAC, glutathione ethyl ester (GEE), and Trolox]. ROS scavengers markedly inhibited the ZFL-induced Ca^2+^ release and the expression of ATF4 and CHOP (Fig. 4a). Furthermore, Mito-TEMPO (a mitochondrial ROS scavenger) also markedly inhibited ZFL-induced Ca^2+^ release and the expression of ATF4 and CHOP (Fig. 4b). We previously reported that HSP70 could inhibit ZFL-induced LMP^19^. Ectopic expression of HSP70 also inhibited ZFL-induced Ca^2+^ release and the expression of ATF4 and CHOP (Fig. 4c). Therefore, our data indicate that LMP-mediated mitochondrial ROS production plays a critical role in ZFL-mediated ER stress via the up-regulation of cytosolic calcium levels.

### ZFL-mediated ER stress enhances death receptor- or anti-cancer drug-induced apoptosis

**Fig. 5:**
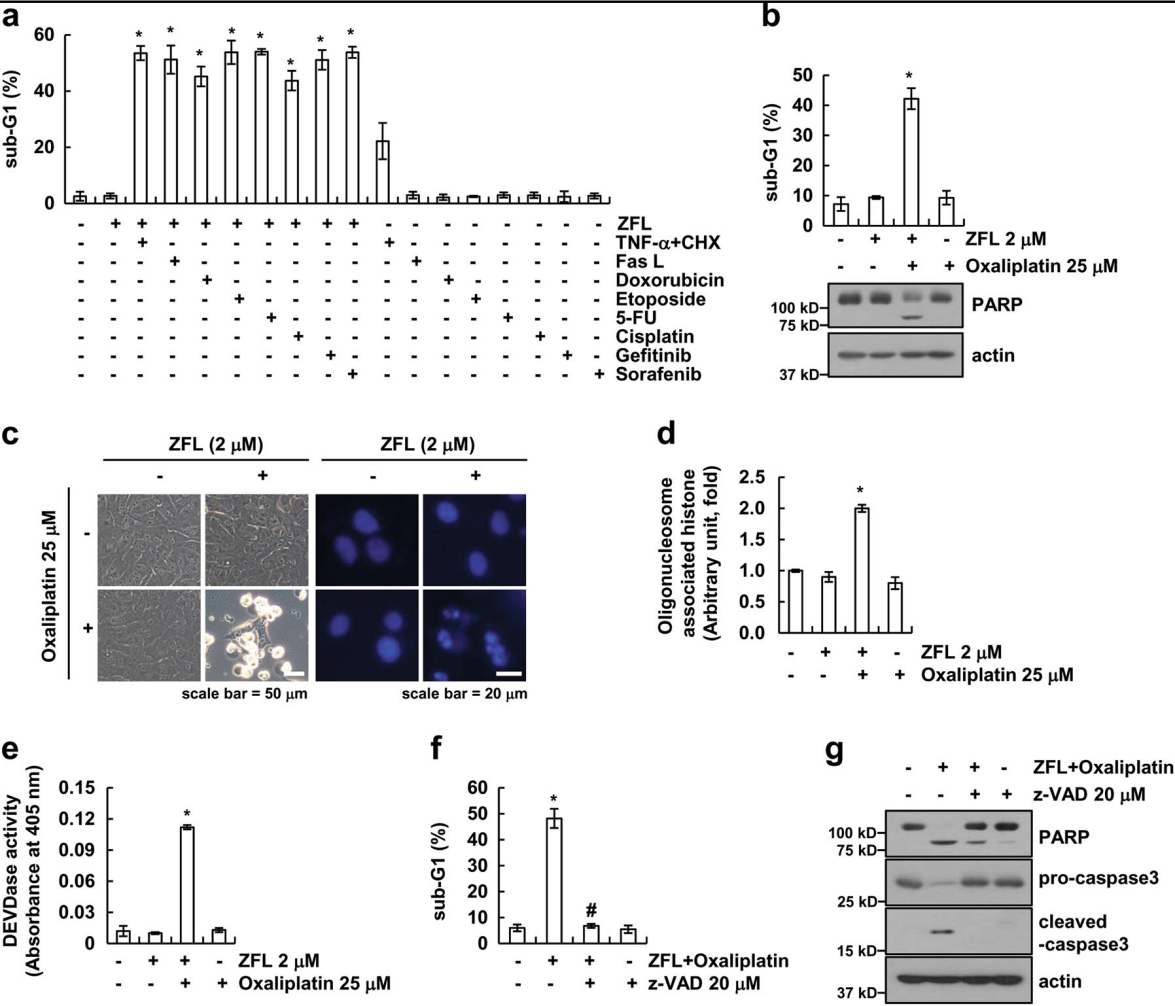
ZFL enhances oxaliplatin-mediated apoptosis. **a** Caki cells were treated with 10 ng/mL TNF-α plus 2.5 μg/mL CHX, 500 ng/mL Fas ligand (Fas L), 1 μM doxorubicin, 3 μg/mL etoposide, 250 μΜ 5-FU, 30 μΜ cisplatin, 0.1 μΜ gefitinib, and 5 μΜ sorafenib in the presence or absence of 2 μM ZFL for 24 h. **b**–**e** Caki cells were treated with 25 μM oxaliplatin in the presence or absence of 2 μM ZFL for 24 h. We analyzed cell morphology using interference light microscopy and nuclei condensation and fragmentation using DAPI staining (**c**). Cytoplasmic DNA fragments (**d**) and caspase activity (**e**) were detected using a kit, as described in the Materials and methods section. **f**, **g** Caki cells were treated with 2 μM ZFL plus 25 μM oxaliplatin in the presence or absence of 20 μM z-VAD-fmk (z-VAD) for 24 h. Flow cytometry was used to detect the sub-G1 fraction, and western blotting was used to detect the protein levels of PARP, pro-caspase-3, cleaved-caspase-3, and/or actin. The values in (**a**, **b**, **d**, **e**, **f**) represent the mean ± SD of three independent samples; *p < 0.01 compared to the control; ^#^*p* < 0.01 compared to ZFL plus oxaliplatin

Previous studies reported that the induction of ER stress enhanced the sensitivity of anti-cancer drugs^27–29^. Therefore, we examined whether ZFL enhances the ligands of death receptors or anti-cancer drug-induced cell death. All tested agents markedly induced apoptosis in ZFL-treated cells (Fig. 5a). However, a sub-lethal dose of a single agent did not induce apoptosis. We chose oxaliplatin for further studies because it is an effective chemotherapeutic drug in several types of cancer. Oxaliplatin alone and ZFL alone did not increase apoptosis, but oxaliplatin plus ZFL markedly induced apoptosis and PARP cleavage (Fig. 5b). Furthermore, oxaliplatin plus ZFL altered cellular morphology and induced chromatin damage of the nucleus (Fig. 5c) and cytoplasmic DNA fragments (Fig. 5d). Combined treatment with oxaliplatin and ZFL markedly increased caspase-3 activation (Fig. 5e), and a pan-caspase inhibitor (z-VAD) attenuated oxaliplatin and ZFL-induced apoptosis as well as the cleavage of caspase-3 and PARP (Fig. 5f, g).

### ER stress plays a critical role in oxaliplatin plus ZFL-induced apoptosis

**Fig. 6:**
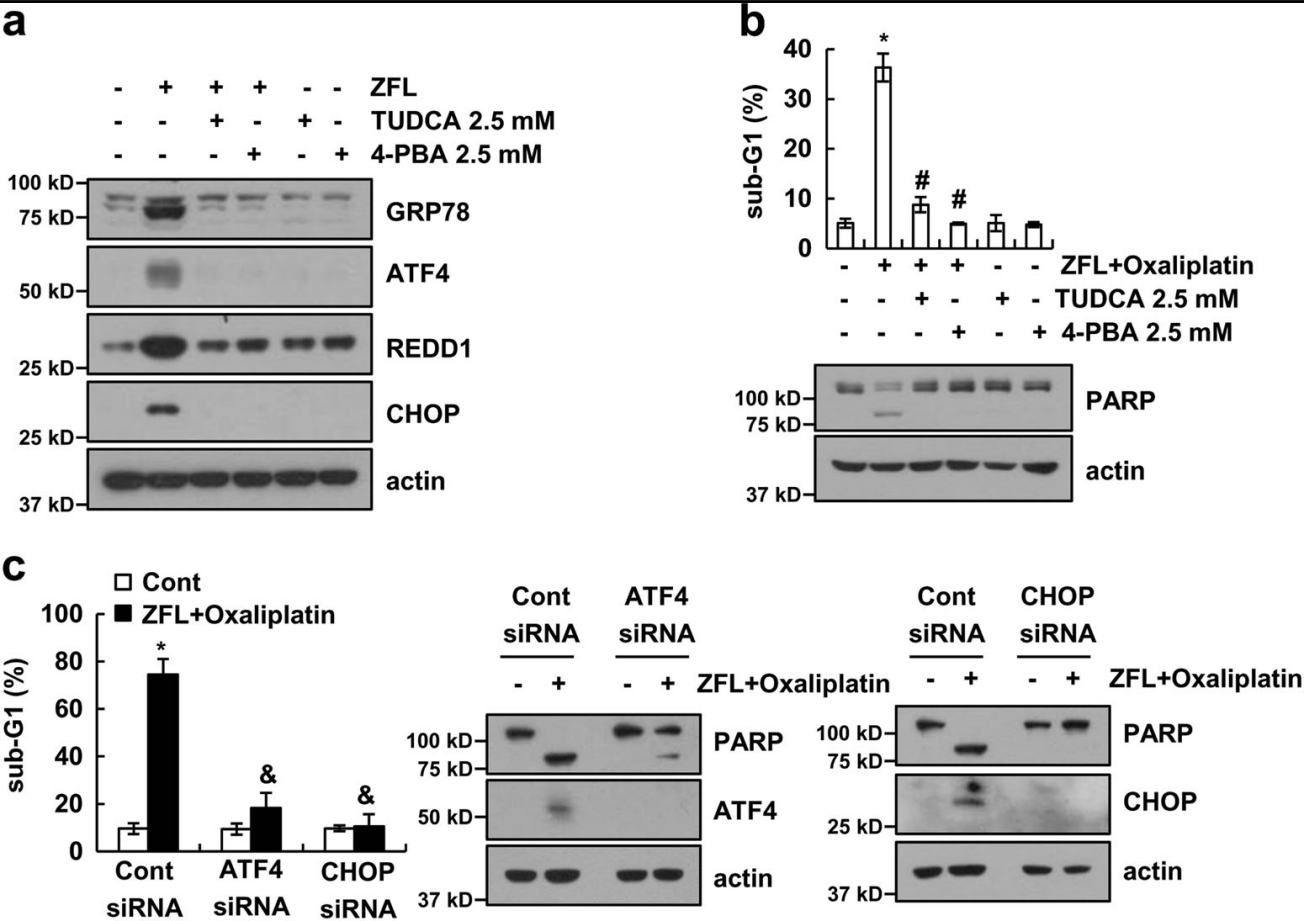
Induction of ER stress is associated with ZFL plus oxaliplatin-induced apoptosis. **a** Caki cells were pre-treated with 2.5 mM TUDCA and 2.5 mM 4-PBA for 30 min, and then 2 μM ZFL was added for 8 h. **b** Caki cells were pre-treated with 2.5 mM TUDCA and 2.5 mM 4-PBA for 30 min and then treated with ZFL plus oxaliplatin for 24 h. **c** Caki cells were transiently transfected with siRNA against the control, ATF4, and CHOP. After 24 h, cells were treated with 2 μM ZFL plus 25 μM oxaliplatin for 24 h. Flow cytometry was used to detect the sub-G1 fraction, and western blotting was used to detect the protein levels of GRP78, ATF4, REDD1, CHOP, PARP, and/or actin. The values in (**b**, **c**) represent the mean ± SD of three independent samples; *p < 0.01 compared to the control; ^#^*p* < 0.01 compared to ZFL plus oxaliplatin; ^&^*p* < 0.01 compared to ZFL plus oxaliplatin-treated control siRNA

Recent studies have reported that chemical chaperones such as tauroursodeoxycholic acid (TUDCA) and 4-phenylbutyric acid (PBA) reduce ER stress^42,43^. Therefore, we investigated the functional significance of ER stress responses under the combined treatment of these chaperones with oxaliplatin and ZFL. TUDCA and 4-PBA markedly inhibited ZFL-induced GRP78, ATF4, REDD1, and CHOP expression (Fig. 6a). Both chemical chaperones also inhibited oxaliplatin plus ZFL-induced apoptosis and cleavage of PARP (Fig. 6b).

The functional role of ATF4 and CHOP in oxaliplatin plus ZFL-induced cell death was investigated by knockdown using siRNA. We found that combined treatment-induced apoptosis and PARP cleavage were markedly attenuated by transfection with ATF4 or CHOP siRNA (Fig. 6c). Our results indicate that ER stress plays a critical role in oxaliplatin plus ZFL-induced apoptotic cell death.

### Oxaliplatin plus ZFL induces apoptosis in other cancer cells

**Fig. 7:**
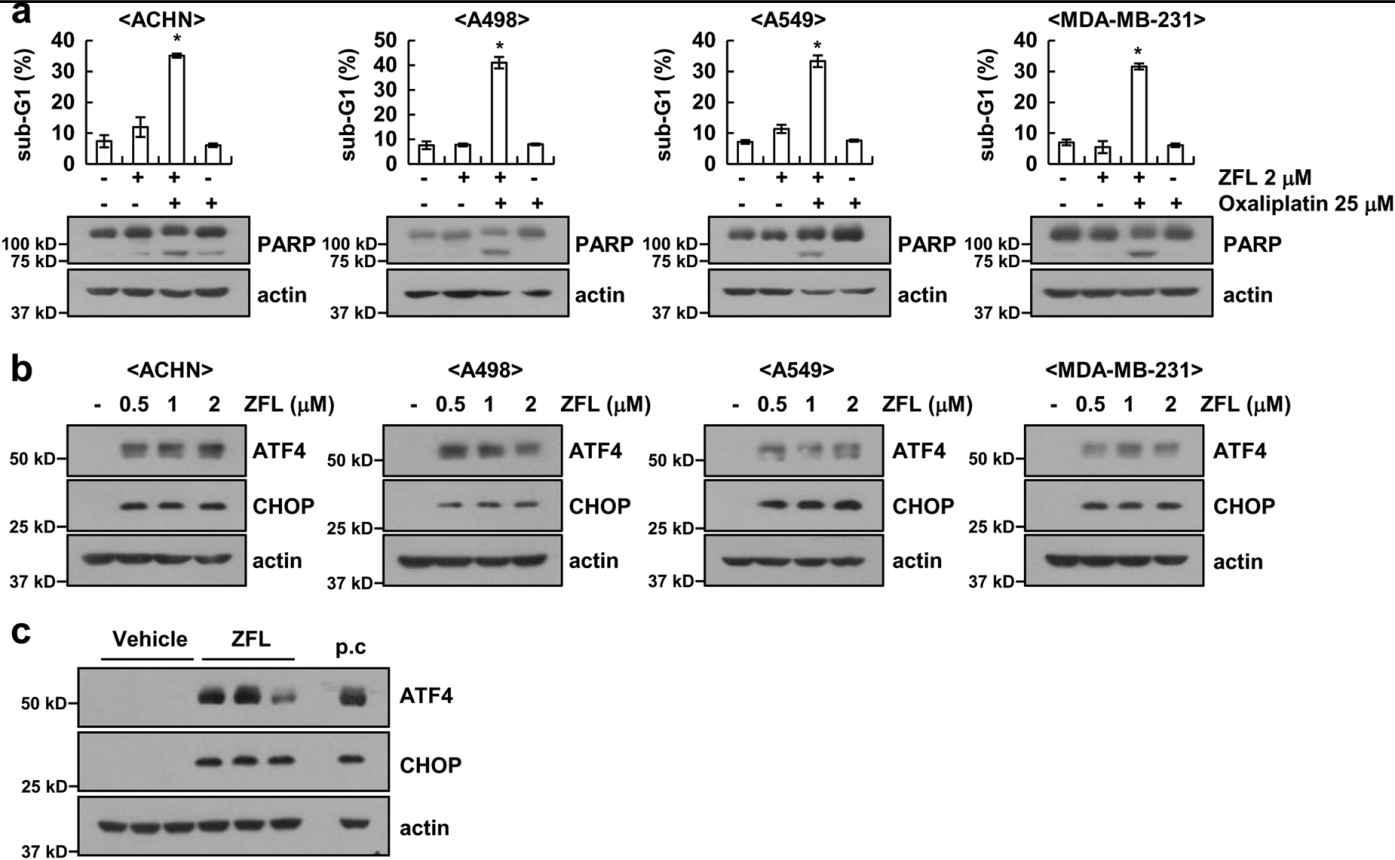
Effects of ZFL on induction of ATF4 and CHOP expression in other cancer cells and in xenograft models. **a** Cancer cells were treated with 25 μM oxaliplatin in the presence or absence of 2 μM ZFL for 24 h. Flow cytometry was used to detect the sub-G1 fraction. **b** Cancer cells were treated with 0.5, 1 or 2 μM ZFL for 8 h. **c** Mice were treated with vehicle or ZFL (5 mg/kg; i.p.) for 21 days. Western blotting was used to detect the protein levels of ATF4, CHOP, and actin. The values in (**a**) represent the mean ± SD of three independent samples; *p < 0.01 compared to the control

**Fig. 8:**
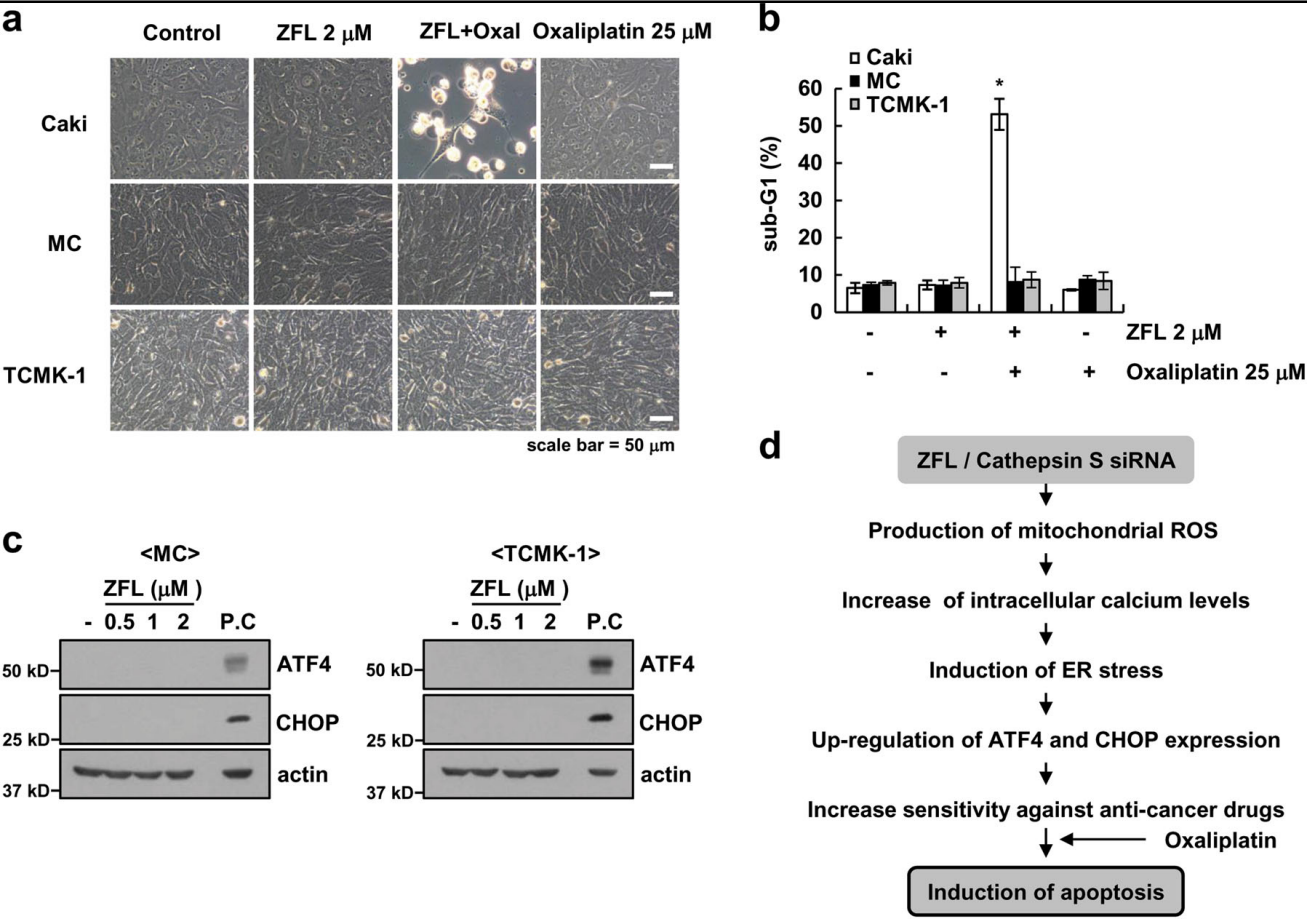
Effects of ZFL on the induction of ATF4 and CHOP expression and oxaliplatin-mediated apoptosis in normal cells. **a**, **b** Caki, mesangial, and TCMK-1 cells were treated with 25 μM oxaliplatin in the presence or absence of 2 μM ZFL for 24 h. Interference light microscopy was used to analyze the cellular morphology (**a**), and flow cytometry was used to measure the sub-G1 fraction (**b**). **c** Mesangial cells and TCMK-1 cells were treated with 0.5, 1, 2 μM ZFL for 8 h (p.c. positive control; ZFL for 8 h in Caki cell). Western blotting was used to detect the protein levels of ATF4, CHOP, and actin. **d** Proposed mechanism for the oxaliplatin-induced apoptosis by ZFL and the down-regulation of cathepsin S. The values in (**b**) represent the mean ± SD of three independent samples; *p < 0.01 compared to the control

We next investigated the effect of oxaliplatin and ZFL on apoptosis in other renal carcinoma cells (A498 and ACHN cells) and other cancer cells (human lung carcinoma (A549) and breast carcinoma (MDA-MB-231)). We found that oxaliplatin plus ZFL induced apoptosis and cleavage of PARP (Fig. 7a) and that ZFL also induced the up-regulation of ATF4 and CHOP in all tested cells (Fig. 7b). Furthermore, we investigated the effect of ZFL on the induction of ER stress using an in vivo xenograft model. Mice bearing tumors were treated with ZFL, and we found that ZFL increased the expression of ATF4 and CHOP (Fig. 7c). However, oxaliplatin plus ZFL did not induce morphological changes or cell death in normal human mesangial cells or normal mouse kidney cells (TCMK-1) (Fig. 8a, b). In addition, ZFL did not increase the expression of ER stress marker proteins in normal cells (Fig. 8c). Therefore, our results indicate that ZFL may selectively sensitize cancer cells to oxaliplatin-induced apoptotic cell death while sparing normal cells.

## Discussion

In the present study, we demonstrated that inhibition of cathepsin S induced cytosolic Ca^2+^ release from the ER, resulting in the induction of ER stress. Furthermore, up-regulation of CHOP and ATF4 expression by ER stress was associated with sensitization to anti-cancer drug-induced apoptosis in ZFL-treated cancer cells (Fig. 8d).

Moderate ER stress usually plays a pro-survival role, returning the cell to homeostasis^44^. However, high loads of UPR and prolonged ER stress induce caspase-mediated apoptosis^44^. Our data indicate that inhibition of cathepsin S by siRNA or ZFL induces the ER stress response but does not induce apoptotic cell death. As shown in Fig. 1d, e, ZFL transiently induced the up-regulation of ER stress marker proteins, but thapsigargin induced and maintained the up-regulation of all proteins for up to 36 h. Moreover, thapsigargin, but not ZFL, induced cell death. Therefore, the transient induction of ER stress by ZFL may affect signaling molecules that induce sensitivity to anti-cancer drugs. In addition, ZFL enhanced oxaliplatin-induced apoptosis in various cancer cell lines, but combined treatment did not increase apoptosis in normal cells (Figs. 7a, 8b). Because ZFL did not induce the expression of ER stress marker proteins in normal cells, the mode of ZFL-induced ER stress may be dependent upon the cell context and cell type.

Deficiency of cathepsin proteins (cathepsin L, E, and S) induces mitochondrial dysfunction^45–47^. We reported that an inhibitor of cathepsin S generated mitochondrial ROS levels by affecting LMP, which is involved in mitochondrial dysfunction^19^. HSP70 was identified as the first survival protein that functions by preventing death-associated LMP^48^. We also previously reported that overexpression of HSP70 inhibited the induction of LMP and markedly blocked ROS production in ZFL-treated cells^19^. In the present study, ectopic expression of HSP70 inhibited Ca^2+^ release and the expression of ATF4 and CHOP in ZFL-treated cells (Fig. 4c). Therefore, ZFL-induced LMP may act as an initial signal, which causes calcium-mediated ER stress. Furthermore, our previous study suggested that ZFL has an anti-cancer effect via the up-regulation of p53 expression. Therefore, we examined the relation between p53 and ER stress. Down-regulation of CHOP or ATF4 by siRNA inhibited ZFL-induced p53 expression (data not shown). Lin et al.^49^ reported that ER stress increases p53 expression at the transcription level through nuclear factor (NF)-κB activation. ER stress inducers (tunicamycin or brefeldin A) induced the nuclear localization of NF-κB, leading to an increase in p53 mRNA expression. The authors suggested that induction of p53 is related to ER stress-induced apoptosis^49^. In addition, CHOP activates NF-κB signaling^50^. CHOP binds to the promoter of peroxisome proliferator-activated receptor-γ (PPARγ), a negative regulator of NF-κB activity, resulting in the suppression of PPARγ expression^50^. Therefore, there is a possibility that inhibition of cathepsin S induces the up-regulation of p53 expression via CHOP-mediated NF-κB activation. Further experiments are warranted to identify the mechanism underlying the ATF4 or CHOP-mediated up-regulation of p53 expression. Previous studies reported that inhibition of cathepsin S induces cell death via the induction of autophagy^8–10^. However, since inhibition of cathepsin S did not induce autophagy in Caki cells^19^, we concluded that ER stress induced by the inhibition of cathepsin S is not related to autophagy in our system. In addition, c-Jun N-terminal kinase (JNK) activation is one of the mechanisms to induce cell death by inhibition of cathepsin S; however, a JNK inhibitor (SP600125) did not reverse ZFL plus oxaliplatin-induced apoptosis (negative data; data not shown). Therefore, ER stress caused by the inhibition of cathepsin S sensitized cells to anti-cancer drug-mediated apoptosis through the modulation of apoptosis-related proteins rather than the induction of autophagy or the activation of JNK phosphorylation.

Both calcium and ROS are cellular signaling molecules, and they can interact to modulate cellular responses. In our study, mitochondrial ROS were found to regulate intracellular calcium levels (Fig. 4b). Previous studies suggest that mitochondrial ROS modulate calcium channel activity in the ER via the following mechanisms. (1) RyRs and IP3Rs have reactive Cys thiols at multiple sites, and thiol oxidation by ROS increases RyR activity. Menshikova and Salama^51^ reported that both reactive disulfides and nitric oxide induce the oxidation of RyRs, resulting in increased release of calcium and increased cytosolic calcium levels. In addition, superoxide and hydrogen peroxides also increase the release of calcium via the oxidation of thiol groups in RyRs^52,53^. (2) ROS decrease the threshold concentration for receptor activation. Hu et al.^34^ reported that hydrogen peroxide and NADPH increase the sensitivity of IP3R via a decrease in the threshold concentration of InsP3-induced intracellular calcium release. (3) ROS may regulate interactions of RyRs-FK506 binding protein (FKBP). The cytoplasmic domain of the RyR acts as a scaffold, and multiple proteins (FKBP, calmodulin, phosphodiesterase, kinases, and phosphatases) bind to this domain, which modulates RyR receptor activity^54^. Among these proteins, FKBP is important for RyR activity^55^, and interactions between FKBP and RyR are modulated by the ROS state. ROS induces FKBP dissociation from RyR, which leads to an increase in calcium release^56^.

Collectively, these results reveal that the inhibition of cathepsin S sensitizes cells to apoptosis induced by various anti-cancer drugs through the calcium-mediated up-regulation of ER stress. Therefore, inhibition of cathepsin S may be an effective strategy for the enhancement of cell sensitivity to anti-cancer drugs.

## References

[1] Driessen C (1999). Cathepsin S controls the trafficking and maturation of MHC class II molecules in dendritic cells. J. Cell Biol..

[2] Pluger EB (2002). Specific role for cathepsin S in the generation of antigenic peptides in vivo. Eur. J. Immunol..

[3] Clark AK, Wodarski R, Guida F, Sasso O, Malcangio M (2010). Cathepsin S release from primary cultured microglia is regulated by the P2X7 receptor. Glia.

[4] Nakagawa TY (1999). Impaired invariant chain degradation and antigen presentation and diminished collagen-induced arthritis in cathepsin S null mice. Immunity.

[5] Fernandez PL (2001). Expression of cathepsins B and S in the progression of prostate carcinoma. Int. J. Cancer.

[6] Flannery T (2003). The clinical significance of cathepsin S expression in human astrocytomas. Am. J. Pathol..

[7] Xu J (2009). Cathepsin S is aberrantly overexpressed in human hepatocellular carcinoma. Mol. Med. Rep..

[8] Chen KL (2012). Targeting cathepsin S induces tumor cell autophagy via the EGFR-ERK signaling pathway. Cancer Lett..

[9] Huang CC, Chen KL, Cheung CH, Chang JY (2013). Autophagy induced by cathepsin S inhibition induces early ROS production, oxidative DNA damage, and cell death via xanthine oxidase. Free Radic. Biol. Med..

[10] Zhang L, Wang H, Xu J, Zhu J, Ding K (2014). Inhibition of cathepsin S induces autophagy and apoptosis in human glioblastoma cell lines through ROS-mediated PI3K/AKT/mTOR/p70S6K and JNK signaling pathways. Toxicol. Lett..

[11] Wang X (2015). Cathepsin S silencing induces apoptosis of human hepatocellular carcinoma cells. Am. J. Transl. Res..

[12] Fan Q (2012). Silencing cathepsin S gene expression inhibits growth, invasion and angiogenesis of human hepatocellular carcinoma in vitro. Biochem. Biophys. Res. Commun..

[13] Gocheva V (2006). Distinct roles for cysteine cathepsin genes in multistage tumorigenesis. Genes Dev..

[14] Flannery T (2006). Cathepsin S expression: an independent prognostic factor in glioblastoma tumours–a pilot study. Int. J. Cancer.

[15] Kos J (2001). Cathepsin S in tumours, regional lymph nodes and sera of patients with lung cancer: relation to prognosis. Br. J. Cancer.

[16] Gormley JA (2011). The role of cathepsin S as a marker of prognosis and predictor of chemotherapy benefit in adjuvant CRC: a pilot study. Br. J. Cancer.

[17] Ward C (2010). Antibody targeting of cathepsin S inhibits angiogenesis and synergistically enhances anti-VEGF. PLoS One.

[18] Burden RE (2012). Inhibition of Cathepsin S by Fsn0503 enhances the efficacy of chemotherapy in colorectal carcinomas. Biochimie.

[19] Seo BR (2017). Inhibition of cathepsin S induces mitochondrial ROS that sensitizes TRAIL-mediated apoptosis through p53-mediated downregulation of Bcl-2 and c-FLIP. Antioxid. Redox Signal..

[20] Logue SE, Cleary P, Saveljeva S, Samali A (2013). New directions in ER stress-induced cell death. Apoptosis.

[21] Verfaillie T, Garg AD, Agostinis P (2013). Targeting ER stress induced apoptosis and inflammation in cancer. Cancer Lett..

[22] Zinszner H (1998). CHOP is implicated in programmed cell death in response to impaired function of the endoplasmic reticulum. Genes Dev..

[23] Guo H (2017). Kaempferol induces hepatocellular carcinoma cell death via endoplasmic reticulum stress-CHOP-autophagy signaling pathway. Oncotarget.

[24] Jang JH, Min KJ, Kim S, Park JW, Kwon TK (2016). RU486 induces pro-apoptotic endoplasmic reticulum stress through the induction of CHOP expression by enhancing C/EBPdelta expression in human renal carcinoma Caki cells. J. Cell. Biochem..

[25] Chen YJ (2013). Sinulariolide induced hepatocellular carcinoma apoptosis through activation of mitochondrial-related apoptotic and PERK/eIF2alpha/ATF4/CHOP pathway. Molecules.

[26] Sanchez-Lopez E (2013). Choline kinase inhibition induces exacerbated endoplasmic reticulum stress and triggers apoptosis via CHOP in cancer cells. Cell Death Dis..

[27] Trivedi R, Maurya R, Mishra DP (2014). Medicarpin, a legume phytoalexin sensitizes myeloid leukemia cells to TRAIL-induced apoptosis through the induction of DR5 and activation of the ROS-JNK-CHOP pathway. Cell Death Dis..

[28] Yoon MJ (2014). Stronger proteasomal inhibition and higher CHOP induction are responsible for more effective induction of paraptosis by dimethoxycurcumin than curcumin. Cell Death Dis..

[29] Gupta SC, Francis SK, Nair MS, Mo YY, Aggarwal BB (2013). Azadirone, a limonoid tetranortriterpene, induces death receptors and sensitizes human cancer cells to tumor necrosis factor-related apoptosis-inducing ligand (TRAIL) through a p53 protein-independent mechanism: evidence for the role of the ROS-ERK-CHOP-death receptor pathway. J. Biol. Chem..

[30] Zeng XC (2004). Hsp70 dynamics in vivo: effect of heat shock and protein aggregation. J. Cell. Sci..

[31] Seo SU, Kim TH, Kim DE, Min KJ, Kwon TK (2017). NOX4-mediated ROS production induces apoptotic cell death via down-regulation of c-FLIP and Mcl-1 expression in combined treatment with thioridazine and curcumin. Redox Biol..

[32] Park YS, Kwon YJ, Chun YJ (2017). CYP1B1 activates Wnt/beta-catenin signaling through suppression of Herc5-mediated ISGylation for protein degradation on beta-catenin in HeLa cells. Toxicol. Res..

[33] Jo Y, Shin DY (2017). Repression of the F-box protein Skp2 is essential for actin damage-induced tetraploid G1 arrest. BMB Rep..

[34] Hu Q (2000). NADPH oxidase activation increases the sensitivity of intracellular Ca2+stores to inositol 1,4,5-trisphosphate in human endothelial cells. J. Biol. Chem..

[35] Um HJ, Park JW, Kwon TK (2011). Melatonin sensitizes Caki renal cancer cells to kahweol-induced apoptosis through CHOP-mediated up-regulation of PUMA. J. Pineal Res..

[36] Clapham DE (2007). Calcium signaling. Cell.

[37] Marks AR (1992). Calcium channels expressed in vascular smooth muscle. Circulation.

[38] Maruyama T, Kanaji T, Nakade S, Kanno T, Mikoshiba K (1997). 2APB, 2-aminoethoxydiphenyl borate, a membrane-penetrable modulator of Ins(1,4,5)P3-induced Ca2+release. J. Biochem..

[39] Xu L, Tripathy A, Pasek DA, Meissner G (1998). Potential for pharmacology of ryanodine receptor/calcium release channels. Ann. N. Y. Acad. Sci..

[40] Vasington FD, Gazzotti P, Tiozzo R, Carafoli E (1972). The effect of ruthenium red on Ca 2+ transport and respiration in rat liver mitochondria. Biochim. Biophys. Acta.

[41] Kuba M, Higure Y, Susaki H, Hayato R, Kuba K (2007). Bidirectional Ca2+coupling of mitochondria with the endoplasmic reticulum and regulation of multimodal Ca2+entries in rat brown adipocytes. Am. J. Physiol. Cell. Physiol..

[42] Xie Q (2002). Effect of tauroursodeoxycholic acid on endoplasmic reticulum stress-induced caspase-12 activation. Hepatology.

[43] Ozcan U (2006). Chemical chaperones reduce ER stress and restore glucose homeostasis in a mouse model of type 2 diabetes. Science.

[44] Wang WA, Groenendyk J, Michalak M (2014). Endoplasmic reticulum stress associated responses in cancer. Biochim. Biophys. Acta.

[45] Petermann I (2006). Lysosomal, cytoskeletal, and metabolic alterations in cardiomyopathy of cathepsin L knockout mice. FASEB J..

[46] Pan L (2012). Cathepsin S deficiency results in abnormal accumulation of autophagosomes in macrophages and enhances Ang II-induced cardiac inflammation. PLoS One.

[47] Tsukuba T (2013). Cathepsin E deficiency impairs autophagic proteolysis in macrophages. PLoS One.

[48] Nylandsted J (2004). Heat shock protein 70 promotes cell survival by inhibiting lysosomal membrane permeabilization. J. Exp. Med..

[49] Lin WC (2012). Endoplasmic reticulum stress stimulates p53 expression through NF-kappaB activation. PLoS One.

[50] Park SH (2010). Endoplasmic reticulum stress-activated C/EBP homologous protein enhances nuclear factor-kappaB signals via repression of peroxisome proliferator-activated receptor gamma. J. Biol. Chem..

[51] Menshikova EV, Salama G (2000). Cardiac ischemia oxidizes regulatory thiols on ryanodine receptors: captopril acts as a reducing agent to improve Ca2+uptake by ischemic sarcoplasmic reticulum. J. Cardiovasc. Pharmacol..

[52] Kawakami M, Okabe E (1998). Superoxide anion radical-triggered Ca2+release from cardiac sarcoplasmic reticulum through ryanodine receptor Ca2+channel. Mol. Pharmacol..

[53] Anzai K (1998). Effects of hydroxyl radical and sulfhydryl reagents on the open probability of the purified cardiac ryanodine receptor channel incorporated into planar lipid bilayers. Biochem. Biophys. Res. Commun..

[54] Zalk R, Lehnart SE, Marks AR (2007). Modulation of the ryanodine receptor and intracellular calcium. Annu. Rev. Biochem..

[55] Richardson SJ (2017). Association of FK506 binding proteins with RyR channels - effect of CLIC2 binding on sub-conductance opening and FKBP binding. J. Cell Sci..

[56] Holmberg SR (1991). Reactive oxygen species modify the structure and function of the cardiac sarcoplasmic reticulum calcium-release channel. Cardioscience.

